# Standardization of D‐dimer reporting in the COVID‐19 era

**DOI:** 10.1002/rth2.12772

**Published:** 2022-09-20

**Authors:** Litao Zhang, Zhenlu Zhang

**Affiliations:** ^1^ Clinical Laboratory Wuhan Asia General Hospital Affiliated to Wuhan University of Science and Technology Wuhan China; ^2^ Laboratory Medicine Wuhan Asia Heart Hospital Wuhan China

**Keywords:** COVID‐19, D‐dimer, laboratory, standardization, thrombosis

D‐dimer test, one of the most common tests for coagulation, plays an important role in management of patients with thrombosis, and its demand has risen sharply during the current global COVID‐19 epidemic. However, some main limitations, including inconsistent reporting units, various thresholds for different assays, lack of standardization, and poor harmonization,^1^ have followed its widespread application and may cause confusion and misinformation.^2^ Recently, a communication from the ISTH Scientific and Standardization Subcommittee (SSC) on Fibrinolysis reported by Bevan and Longstaff described a potential method to generate a stable standard material for D‐dimer.^3^ This is quite important for standardization of D‐dimer testing; however, future investigations are still needed for confirmation. What can we do to improve the standardization of D‐dimer results before achieving the D‐dimer assay standardization? Based on recent evidence and the authors' experience, we have two proposals in an unofficial position.

First, reporting the D‐dimer ratio (DDR) would be a step forward in standardizing D‐dimer reporting. DDR means the ratio of the D‐dimer value to the upper limit of the normal range (ULN) for the current D‐dimer assay (DDR = D‐dimer/ULN; e.g., DDR = 2.0 for a D‐dimer = 1000 ng/ml with 0–500 ng/ml of normal range). DDR can show directly a proportional level of D‐dimer elevation, and is independent of type of unit used, and also accounts for the cutoff value used. This is a simple and helpful transformation to improve the comparability among various D‐dimer detection methods. We carried out a pilot study to test D‐dimer values of 12 samples using four common commercial D‐dimer assays simultaneously (Table 1). The original D‐dimer values from the same sample analyzed with four different assays showed a composite of ~500‐fold differences, compared to the differences of no more than 2‐fold in DDR values (D‐dimer value vs. DDR, 513.8 ± 97.4 vs 1.46 ± 0.21; see Table 1 for calculation details). Apparently, Reporting DDR could potentially remove the heterogeneity of the D‐dimer results reported from different assays.

**TABLE 1:** D‐dimer and DDR results of 12 samples on four commercial assays

	D‐dimer value					D‐dimer ratio				
	Assay 1^a^	Assay 2^b^	Assay 3^c^	Assay 4^d^	Maximum difference^e^	Assay 1	Assay 2	Assay 3	Assay 4	Maximum difference
ULN	243	0.5	1	0.5						
Report unit	ng/mL	mg/L	μg/ml	μg/ml						
Express unit	DDU	FEU	DDU	FEU						
Sample 1	130	0.32	0.60	0.27	481.5	0.53	0.64	0.6	0.54	1.21
Sample 2	132	0.25	0.96	0.34	528.0	0.54	0.50	0.96	0.68	1.92
Sample 3	153	0.26	0.78	0.37	588.5	0.63	0.52	0.78	0.74	1.50
Sample 4	254	0.64	1.02	0.64	396.9	1.04	1.28	1.02	1.28	1.25
Sample 5	755	2.05	2.87	1.97	383.2	3.11	4.10	2.87	3.94	1.43
Sample 6	967	2.52	3.83	2.55	383.7	3.98	5.04	3.83	5.10	1.33
Sample 7	1030	1.82	3.70	1.59	647.8	4.24	3.64	3.70	3.18	1.33
Sample 8	1525	3.07	5.33	3.34	496.7	6.28	6.14	5.33	6.68	1.25
Sample 9	2091	4.67	5.97	4.01	521.4	8.61	9.34	5.97	8.02	1.56
Sample 10	2772	6.18	7.40	4.97	557.7	11.41	12.36	7.40	9.94	1.67
Sample 11	2891	5.83	8.07	5.94	495.9	11.90	11.66	8.07	11.88	1.47
Sample 12	5095	7.44	13.38	8.01	684.8	20.97	14.88	13.38	16.02	1.57
Total					513.8 ± 97.4					1.46 ± 0.21

Abbreviations: DDU, D‐dimer unit; FEU, fibrinogen equivalent unit; ULN, the upper limit of the normal range.

^a^ Assay 1, Werfen ACL‐TOP 750 with HemosIL D‐Dimer HS reagent;

^b^ Assay 2, Sysmex CS5100 with Siemens Innovance D‐dimer reagent;

^c^ Assay 3, Sekisui CP3000 with Nanopia D‐dimer reagent;

^d^ Assay 4, Stago STA‐R MAX with STA Liatest D‐Di reagent.

^e^ Maximum difference means that the maximum value of four results from the same sample divided by the minimum; e.g., Sample 12: 5095 divided by 7.44 is 684.8 for D‐dimer value, and 20.97 divided by 13.38 is 1.57 for D‐dimer ratio.

More importantly, DDR has been used and accepted by more and more clinicians in clinical practice, especially in the management of COVID‐19. D‐dimer was measured in 28 hospitals from five countries in the RAPID study,^4^ a randomized controlled trial that compared the effects of therapeutic heparin with prophylactic heparin among moderately ill patients with COVID‐19. The investigators employed D‐dimer ratios to make the results comparable across sites since they used different types of D‐dimer assays. Similarly, another large multicenter clinical trial (ATTACC, ACTIV‐4a, and REMAP‐CAP) performed at 121 sites in nine countries to evaluate anticoagulation with heparin in patients with COVID‐19 also elected to use the DDR instead of absolute D‐dimer values to stratify the patients in the study cohorts.^5^, ^6^

Second, confusion exists between the fibrinogen equivalent unit (FEU) and D‐dimer unit (DDU), which the manufacturers use to report D‐dimer levels based on the molecular weight of fibrinogen and D‐dimer, respectively.^7^, ^8^ The following points further expand on the difference and difficulties pertaining to FEU and DDU.

1. The only difference between the two units is a difference in molecular weight but has resulted in different normal reference range cutoffs between medical centers with resultant confusion and uncertainty in medical professionals as discussed above.^2^ An official communication from the ISTH SSC on fibrinolysis (Thachil J et al.) recommended that “a standardized measuring units should be used for reporting patient results and suggested FEUs either in μg/L or mg/L.”^8^
2. Age‐adjusted D‐dimer cutoffs have been demonstrated to effectively improve the rule‐out performance of the D‐dimer tests in elderly patients with suspected venous thrombosis. Guidelines propose that an age‐adjusted D‐dimer cutoff value instead of fixed cutoff values^9^ should be implemented. The age‐adjusted D‐dimer cutoff calculation is “Age × 10 mg/L,” for patients older than 50 years. A couple of studies in this regard used DDUs.^10^, ^11^
3. There is evidence that more laboratories are reporting D‐dimers as FEUs versus DDUs,^12^ which further supports migration toward reporting D‐dimers in FEU units. It is in this background that we propose exclusive use of FEUs instead of DDUs to report D‐dimer results.

Such a change in the units of D‐dimer reporting will not be without stumbling blocks. For example, the manufacturers might be hesitant to change product information sheets that have been cleared or approved for use by regulatory authorities. Lippi et al.^12^ proposed that international standardization societies such as the ISTH or the International Federation of Clinical Chemistry and Laboratory Medicine should take the lead in pursuing the challenging undertaking of standardization of the D‐dimer results reporting.^12^ The authors are of the opinion that consistent reporting of D‐dimer tests in FEU will be welcomed by clinicians, research units, standardization societies, and medical educators.

In conclusion, it might be the right time to act to improve the standardization of D‐dimer reporting and help laboratories and clinicians better use the D‐dimer tests.

## AUTHOR CONTRIBUTIONS

LZ and ZZ drafted the manuscript.

## FUNDING INFORMATION

This work was supported by the Hubei Provincial Nature Science Foundation of China (2020CFB865) and 2020 Wuhan Young & Middle‐age Medical Backbone Training Programme.

## RELATIONSHIP DISCLOSURE

The authors declare that they have no conflicts of interest regarding this article.
